# Emerging role of long non-coding RNA MALAT1 related signaling pathways in the pathogenesis of lung disease

**DOI:** 10.3389/fcell.2023.1149499

**Published:** 2023-05-11

**Authors:** Jun Liu, Md Khadem Ali, Yuqiang Mao

**Affiliations:** ^1^ Department of Thoracic Surgery, Shengjing Hospital of China Medical University, Shenyang, China; ^2^ Devission of Pulmonary, Allergy and Critical Care Medicine, School of Medicine, Stanford University, Stanford, CA, United States; ^3^ Clinical Skills Practice Teaching Center, Shengjing Hospital of China Medical University, Shenyang, China

**Keywords:** MALAT1, asthma, COPD, COVID-19, acute respiratory distress syndrome, lung cancer

## Abstract

Long non-coding RNAs (lncRNAs) are endogenously expressed RNAs longer than 200 nt that are not translated into proteins. In general, lncRNAs bind to mRNA, miRNA, DNA, and proteins and regulate gene expression at various cellular and molecular levels, including epigenetics, transcription, post-transcription, translation, and post-translation. LncRNAs play important roles in many biological processes, such as cell proliferation, apoptosis, cell metabolism, angiogenesis, migration, endothelial dysfunction, endothelial-mesenchymal transition, regulation of cell cycle, and cellular differentiation, and have become an important topic of study in genetic research in health and disease due to their close link with the development of various diseases. The exceptional stability, conservation, and abundance of lncRNAs in body fluids, have made them potential biomarkers for a wide range of diseases. LncRNA MALAT1 is one of the best-studied lncRNAs in the pathogenesis of various diseases, including cancers and cardiovascular diseases. A growing body of evidence suggests that aberrant expression of MALAT1 plays a key role in the pathogenesis of lung diseases, including asthma, chronic obstructive pulmonary diseases (COPD), Coronavirus Disease 2019 (COVID-19), acute respiratory distress syndrome (ARDS), lung cancers, and pulmonary hypertension through different mechanisms. Here we discuss the roles and molecular mechanisms of MALAT1 in the pathogenesis of these lung diseases.

## Background

Lung diseases, such as asthma, chronic obstructive pulmonary diseases (COPD), lung cancer, Coronavirus Disease 2019 (COVID-19), acute respiratory distress syndrome (ARDS), idiopathic pulmonary fibrosis (IPF), and pulmonary hypertension (PH) are one of the major causes of death in the world. The effective therapies for lung diseases are limited. There is an urgent unmet need for developing effecting novel therapies for the diseases. To identify novel treatment or targeted interventions, understanding the molecular mechanisms of the signaling pathways involved in the pathogenesis of the disease is still required.

Long non-coding RNAs (lncRNAs) are RNAs longer than 200 nucleotides that lack the ability to code for proteins (they lack functional open reading frames) (Kung et al., 2013). According to GENCODE consortium (Version 42 and M31), 19,933 and 14,764 lncRNAs compared to 19,379 and 21,657 protein-coding genes have been annotated in human and mouse, respectively. Although lncRNAs do not make proteins, they regulate a range of biological processes, such as cell proliferation, apoptosis, migration, angiogenesis, and metabolism (Zhang et al., 2019). LncRNAs act as molecular decoys, scaffolding in signaling transduction, and guiding ribonucleoprotein complexes (Li et al., 2013). LncRNAs plays a significant role in various diseases, such as cancers, cardiovascular and lung diseases (Jiang et al., 2019; Fang et al., 2020). A growing body of evidence suggest that lncRNAs plays a significant role in the onset and progression of lung disease (Groot et al., 2018).

One of the most extensively studied lncRNA is metastasis-associated lung adenocarcinoma transcript 1 (MALAT1, ENSG00000251562), located in human chromosome 11q13.1 (mouse chromosome 19qA). MALAT1 is also known as nuclear-enriched abundant transcript 2 (NEAT2, originally described to be associated with non-small cell lung cancer (Ji et al., 2003). MALAT1 is a nuclear enriched and highly conserved lncRNA that is the most abundantly expressed lncRNA in cells and tissues (Hutchinson et al., 2007). The mouse MALAT1 is 6.7 kb, but human MALAT1 is approximately 8.7 kb in length (Djebali et al., 2012).

At the molecular level, MALAT1 has been reported to play a role in regulating pre-mRNA splicing (Hutchinson et al., 2007; Tripathi et al., 2010), transcription (Arun et al., 2020), and post-transcriptional modifications, such as 5-methylcytosine (Amort et al., 2017) and N^6^-methyladenosine (Liu et al., 2017). In addition, MALAT1 can act as a competing endogenous RNA or miRNA sponge to sequester miRNAs and regulate gene expression. MALAT1 has been shown to be aberrantly expressed and have prognostic and therapeutic significance in different cancers, including pancreatic cancer, lung cancer, breast cancer, colorectal cancer, gastric cancer, nasopharyngeal carcinoma, hepatocellular carcinoma, osteosarcoma (Sun and Ma, 2019). A growing body of evidence reveal that MALAT1 plays a significant role in lung diseases, such as asthma, COPD, COVID-19, IPF and PAH. Here, we summarize the roles and molecular mechanisms, therapeutic and biomarker potential of MALAT1 in lung diseases.

## Role of MALAT1 in lung homeostasis and pathophysiology

Lung homeostasis refers to the balance of physiological processes and cellular activities necessary to maintain the normal structure and function of the lungs. This includes the proper functioning of the airways, alveoli, blood vessels, and other components of lung tissue. Key processes involved in lung homeostasis include gas exchange, immune defense, airway clearance, and proper cell growth and differentiation. The airway plays a vital role in regulating lung fluid balance, removing inhaled agents, attracting, and activating inflammatory cells, and maintaining airway smooth muscle function. The lung alveoli are the basic functional units of the gaseous exchange of oxygen and carbon dioxide between inhaled air and the bloodstream. Under normal circumstances, the airway epithelium forms a crucial tissue barrier and serves as the primary defense against both external and internal challenges (Knight and Holgate, 2003; Crystal et al., 2008). Exposure to toxic stimuli can cause damage to lung epithelial cells, resulting in the breakdown of the epithelial barrier and airway injury (Shaykhiev et al., 2011). This damage to the epithelium triggers the activation of various types of immune cells, including macrophages, dendritic cells (DCs), and others, which further contributes to the inflammatory immune response (Fujita et al., 2015). Proper lung homeostasis is crucial to maintain proper respiratory function and overall health. It ensures that the lungs can effectively exchange oxygen and carbon dioxide while also protecting against harmful pathogens, irritants, and toxins. Disruptions in lung homeostasis can lead to respiratory diseases, such as asthma, COPD, and lung cancer, which can significantly impact quality of life and mortality rates. Therefore, maintaining lung homeostasis is essential for optimal respiratory health.

**FIGURE 1:**
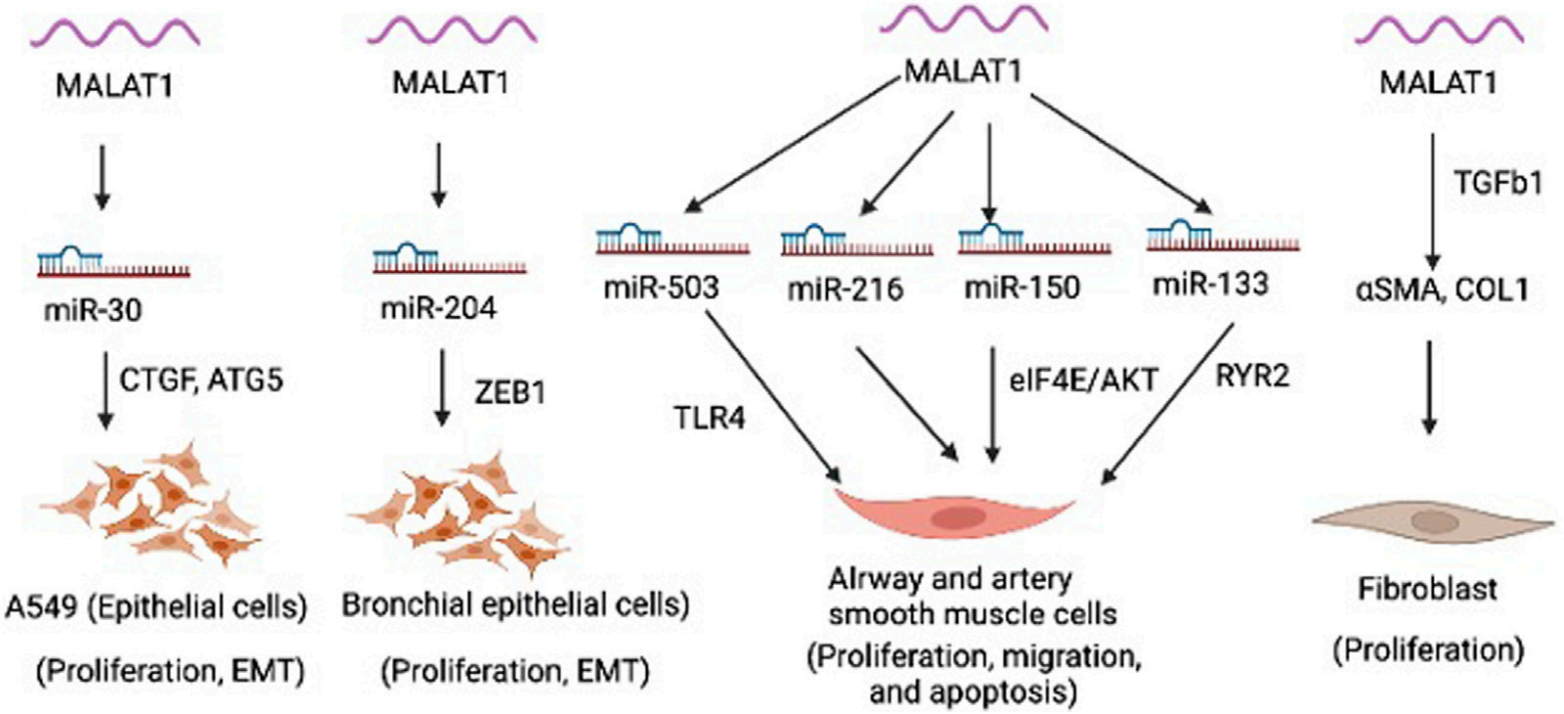
Function of Malat1 in different lung cells.

MALAT1 is expressed in various cell types within the lung, including epithelial cells, smooth muscle cells, fibroblasts, and immune cells. MALAT1 is involved in regulating various cellular processes, such as proliferation, migration, apoptosis, epithelial mesenchymal transition (EMT) (Figure 1). MALAT1 has been implicated in several aspects of lung homeostasis and dysregulation of MALAT1 is involved in the development and progression of different lung diseases, such as asthma, COPD, and lung cancer (Lai et al., 2022). Silencing of MALAT1 in airway smooth muscle cells using siRNA resulted in decreased cell viability, migration and invasion and induced apoptosis as evidenced by upregulated Caspase 3, Bax, E-cadherin and downregulated N-cadherin, Bcl-2 and β-catenin (Huang et al., 2021). MALAT1 was shown to regulate the function of airway smooth muscle cells through the modulation of microRNA-216a (Huang et al., 2021). Another study found that MALAT1 expression in ASMCs increased with PDGF-BB treatment, but knockdown of Malat1 reduced PDGF-BB-induced proliferation and migration (Lin et al., 2019). Malat1 directly targeted miR-150, inhibition of which promoted PDGF-BB-induced proliferation and migration, which was reversed by Malat1 overexpression. miR-150 targets eIF4E, and eIF4E knockdown and Akt inhibitor GSK690693 inhibited PDGF-BB-induced proliferation and migration (Lin et al., 2019). Malat1 was found to operate as a ceRNA for miR-150, derepressing eIF4E expression and activating the Akt pathway, facilitating PDGF-BB-induced ASMC proliferation and migration (Lin et al., 2019). Yang and Wang demonstrated that silencing MALAT1 provides protection against injury to bronchial/tracheal smooth muscle cells by regulating the microRNA-133a/ryanodine receptor 2 axis (Yang and Wang, 2021). Li *et al.*, showed that MALAT1 expression increases in AECs co-cultured with dendritic cells (DCs), but down-regulating MALAT1 expression in this co-culture system leads to increased expression of maturity surface markers (CD86, CD80, and MH class II), production of inflammatory cytokines (TNF-α, IL-6, and IFN-γ), and secretion of chemokines (CXCR2 and CXCR4) in DCs (Li et al., 2018a). Furthermore, suppression of MALAT1 was shown to reduce apoptosis and promotes cell viability in DCs co-cultured with AECs (Li et al., 2018a). These findings suggest that MALAT1-mediated crosstalk between AECs and DCs affects DC maturation, and apoptosis, chemotaxis, and cytokine production. MALAT1 was also shown to promote the proliferation and migration of pulmonary artery smooth muscle cells via modulating mir-503/Toll-Like Receptor 4 (TLR4) Signal Axis (He et al., 2020). Furthermore, as mentioned above, epithelial cells in the respiratory system act as a crucial barrier to protect the internal tissues from the environment. They regulate gas exchange, maintain hydration, and defend against toxins, irritants, and pathogens. MALAT1 was shown to regulate the EMT process in alveolar epithelial cells (A549) by binding to miR-30c-5p and consequently influencing downstream targets such as CTGF, ATG5, and autophagy (Xia et al., 2023). In this study, the author elucidated fibrosis mechanism in silicosis and offered new therapeutic targets. a biological process by which epithelial cells undergo a series of molecular changes that enable them to acquire mesenchymal properties, such as increased motility and invasiveness. This process is essential for embryonic development, tissue repair, and wound healing, but it can also contribute to cancer progression and metastasis. During EMT, epithelial cells lose their characteristic morphology and polarity, and acquire a spindle-like shape and increased motility. Another study showed that MALAT1 mediates PM2.5-induced EMT through miR-204/ZEB1 pathway in bronchial epithelial cell lines (HBE and BEAS-2B) (Luo et al., 2019). These findings suggest that Malat1 may present a new target to modulate smooth muscle cells and epithelial cells in the lung to limit pulmonary vascular and airway remodeling in lung disease.

## Role of MALAT1 in lung disease

Respiratory diseases, such as asthma, COPD, lung cancer, IPF, and CF are among the leading causes of mortality and morbidity, and they represent a major socio-economic burden worldwide (Xie et al., 2020). More specifically, asthma affects over 300 million people in the world and around 14% of all children suffer from the disease (Pearce et al., 2007; Fergeson et al., 2017). IPF, while less common, is a progressive disease that leads to shortness of breath, pulmonary embolisms, pneumonia, and other deadly conditions. Emerging studies suggest that lncRNA MALAT1 plays a significant role in the pathogenesis of lung diseases. In the following sections, we will discuss the involvement of MALAT1 in the pathogenesis of lung diseases, especially asthma, COPD, IPF, ARDS, and PAH.

## MALAT1 and asthma

Asthma is characterised by features, such as cough, shortness of breath, wheezing and chest pain (Mattiuzzi and Lippi, 2020). The National Heart, Lung, and Blood Institute defines asthma as a chronic disease which affects lung airways, and most asthmatic patients may have narrowed and inflamed airways, and obstructed breathing. The researchers have added that pollen, virus infections and exercise can induce or worsen asthma symptoms (Ramsahai et al., 2019). Asthma is no respecter of age as according to the National Health Service; people of all ages can acquire this lung disease. The Centres for Disease Control and Prevention has made it clear that there is at least one asthmatic patient in a group of thirteen in the United States. Patient death is imminent because of failure in timely treatment of symptoms (Yan et al., 2019). Hence, insights into some of the underlying aberrant molecular mechanisms would be helpful in dealing with asthma more effectively.

It is evident that MALAT1 plays significant roles in the asthma pathogenesis and progression, and the work by Huang *et al.* is a typical example (Huang et al., 2021). Using preclinical asthmatic rat models and with the aim to probe the effects of MALAT1 on airway smooth muscle cells in asthma using various biochemical and molecular techniques, the researchers reported that asthma was associated with upregulated MALAT1 and downregulated microRNA-216a, a target of MALAT1 through inhibition. Essentially, downregulated MALAT1 and/or upregulated microRNA-216a significantly elevated apoptosis while cell proliferation, migration and invasion saw tremendous reductions (Huang et al., 2021). In another study, RT-qPCR analysis on blood samples revealed an upregulated MALAT1 in patients with asthma compared to healthy controls (Liang and Tang, 2020). In addition, dysregulated T-cell receptor signalling pathway was found to be a major hallmark in eosinophilic asthma patients (Zhu et al., 2018). However, the only concern, at least per what we could identify, which may present as a limitation to their study, was the small sample size used (n = 9 for eosinophilic asthma patients and n = 3 for healthy controls).

A recent study has reported aberrant proliferation and smooth muscle layer thickening at where airways are located, in lung tissues of newborn rat models of asthma (Yang and Wang, 2021). Using RT-qPCR, the authors discovered a significant upregulation of MALAT1 in the tracheal tissues of rats. The authors also observed synchronous changes in cell apoptosis and secretion of inflammatory factors, such as interleukin-6, tumour necrosis factor alpha, and interleukin-1β, or ryanodine receptor 2 (RyR2) level when MALAT1 was either silenced or overexpressed in the bronchial and tracheal smooth muscle cells. However, changes in miR-133a level with MALAT1 occurred at unrelated times. The authors added that since MALAT1 drives apoptosis in bronchial and tracheal smooth muscle cells, and either miR-133a upregulation or RyR2 silencing can partly reverse inflammation, the knockdown of MALAT1 can mitigate bronchial and tracheal smooth muscle cell injury through the regulation of miR-133a/RyR2 (Yang and Wang, 2021). This study proposes a mechanism by which MALAT1 is involved in asthma in newborn rats, which is an intriguing finding. When taken together with the three previous studies, it becomes evident that MALAT1 plays a crucial role in the pathogenesis of asthma, as demonstrated through preclinical and clinical investigations.

## MALAT1 and IPF

Termed as the most occurring form of permanent interstitial pneumonia (Wang et al., 2020), IPF is a lung disease with no clear etiology. The major hallmark features of IPF are characterized by extensive lung architectural remodeling and subsequent chronic lung malfunction. IPF has an average mortality rate of 4 years, and there is currently no effective therapy available to treat the disease (Raghu et al., 2011; Guiot et al., 2017; Guiot et al., 2018). The prevalence of IPF is estimated to range from 14 to 28 cases per 100,000 in the United States, and 1 to 23 cases per 100,000 in Europe (Nalysnyk et al., 2012). The disease is age and sex-related, with men being more susceptible than women (Nalysnyk et al., 2012). Aside the lack of effective therapeutic intervention, there are other challenges which include unclear physiopathology, lack of a simple-but-effective diagnosis (currently only requires multidisciplinary methods) and a nonspecific radiological image (Poulet et al., 2020).

While the major regulatory genes associated with IPF onset and progression are not clearly known, recent arising evidence shows that MALAT1 is a major regulator of IPF pathogenesis and one of such evidence is the study conducted by Wang et al. (2020). In detail, the authors main aim was to investigate the major roles of lncRNAs, including MALAT1, and transcription factors, in the pathogenesis of IPF using blood samples from human subjects. To achieve this, the approach involved analysis of three Gene Expression Omnibus (GEO) dataset-derived gene expression profiles; GSE 2052, GSE44723, and GSE24206. While hypergeometric testing revealed that several genes were found to relate to ubiquitin-mediated proteolysis, spliceosome, and cell cycle in both IPF patients and healthy controls, MALAT1 was one of the only three expressed genes that differed in peripheral blood samples from the two groups, as measured by qRT-PCR. While this information provides great insight into the involvement of MALAT1 in IPF pathogenesis, the authors have also highlighted several limitations. For instance, although the genes associated with IPF pathogenesis were identified through dataset integration and bioinformatics, no casual molecular and histological investigations of the direct role of MALAT1 in IPF were conducted to support their findings. The authors also acknowledged small sample sizes and imbalances in the datasets as a limitation. Lastly, although the authors successfully confirmed gene expression levels in their clinical investigations, no *in vivo* or *in vitro* loss- and gain-of-function experiments were conducted to facilitate further functional verification of the genes (Wang et al., 2020).

## MALAT1 and ARDS

ARDS is known to be a critical type of acute lung injury, which is distinguished by intense inflammatory reactions that may result in cellular and tissue damage and mortality (Wang et al., 2019a). While the role of MALAT1 in ARDS has still not been completely found, various studies are tirelessly trying to unlock this puzzle. For example, MALAT1, as detected by qRT-PCR, was found to be overexpressed in plasma and peripheral blood mononuclear cells (PBMCs) of ARDS patients relative to healthy controls. In addition, there was an interaction between MALAT1 and miR-425, which in turn resulted in the protection of the expression of phosphatase and tensin homolog (PTEN) in A549 and HFL-1 cells. Lastly, it was discovered that the delivery of miR-425 by exosomes into A549 and HFL-1 cells in ARDS patients was minimal compared to that delivered in healthy controls. The researchers concluded that MALAT1 is upregulated amidst downregulated miR-425 in ARDS, signifying the involvement of MALAT1 in disease pathogenesis (Wang et al., 2019a). A major limitation identified was the lack of the detection of levels of MALAT1 in pathological tissues. Though just a couple of studies detailing the evidence of MALAT1’s involvement in ARDS have been stated in this review, other researchers (Juan et al., 2018; Huang and Zhao, 2019; Yao et al., 2020) have undertaken similar studies and interested readers can also have a look at those.

## MALAT1 and COPD

COPD is a chronic lung inflammatory disease characterized by airflow obstruction in the lung. COPD accounts for the fourth leading cause of death globally (Vogelmeier et al., 2017). The leading risk factor for COPD is smoking, while quitting smoking early on could significantly slow down or even reverse the progression of lung function reduction (Damkjaer et al., 2021). Currently, there is no cure for COPD, but treatment control the symptoms and slow the progression of the disease.

A growing body of evidence suggests that MALAT1 plays a significant role in COPD pathogenesis. A previous study by Hu *et al.*, measured expression levels of six selected lncRNAs based on their involvement in different lung diseases (HOTAIR, HULC, MEG3, NEAT1, UCA1, and MALAT1) by RT-qPCR in the lung tissues collected from three pairs of age-matched COPD and non-COPD individuals (Hu et al., 2020). Among the six lncRNAs, lncRNAs MALAT1 and MEG3 showed a significant upregulation in COPD lung tissues. The authors then further confirm MALAT1 and MEG3 expression in a second cohort consisting of 10 pairs of age-matched COPD and non-COPD individuals and found that lncRNA MALAT1 is consistently upregulated in COPD lung tissues when compared with non-COPD lung tissues. Silencing of MALAT1 with siRNA in human fetal lung fibroblasts treated with TGFb showed a decrease in cell viability and reduction in protein expression of two mesenchymal markers, α-SMA and fibronectin. Mechanistically, the authors demonstrated that the α-SMA and fibronectin expression occurred independently of mTORC1, because p-S6K1 protein expression was not affected following MALAT1 silencing, which further warrants futures studies to investigate this mechanism in COPD.

By comparing MALAT1 expression in the blood of 120 healthy subjects, 120 stable COPD patients, and 120 acute exacerbation COPD patients, Liu *et al.*, observed higher expression of MALAT1 in acute exacerbation COPD patients compared to stable COPD patients and healthy subjects (Liu et al., 2020). The ROC curve analysis revealed that MALAT1 expression discriminates from acute exacerbation COPD patients from the healthy subjects (sensitivity and specificity 99.2% and 83.3%, respectively) and the stable COPD patients (64.2% and 83.3%, respectively), indicating that MALAT1 could be used as a biomarker in COPD. The authors also found a significant positive correlation between expression of MALAT1 and GOLD stage and inflammatory cytokines, such as IL‐1β, IL‐6, IL‐17, IL‐8, IL‐23 and TNF‐α) in both patients with acute exacerbation and stable cohorts, indicating MALAT1 could induce inflammation and inappropriate immune response in COPD. The authors also showed a significant negative correlation of MALAT1 expression with its targets miR‐146a, miR‐125b, and miR‐203 in acute exacerbation COPD patients and a reverse correlation with miR‐146a and miR‐125b in stable COPD patients. Importantly, acute exacerbation COPD patients had the lowest levels of miR-125b, miR-146a, miR-133, and miR-203, followed by in stable COPD patients and HCs. In both stable and acute exacerbation COPD patients, miR-125b, miR-146a, miR-133, and miR-203 were inversely linked with inflammation and GOLD stage. According to these findings, MALAT1 and its microRNA targets may be useful in helping predict the severity of acute exacerbations and the progression of COPD. There are, however, several limitations to these studies. Firstly, it is unclear how MALAT1 contributes to acute exacerbations of COPD. Secondly, acute exacerbation and stable COPD patients were not investigated for the effect of MALAT1 expression on short- and long-term prognosis. Finally, these findings may result in bias since blood samples were collected within 24 h of enrollment of acute exacerbation COPD patients, however, there was a wide range of time intervals between the onset of acute exacerbation COPD and hospital admissions.

## MALAT1 and PAH

PAH is a serious, life-threatening progressive medical condition characterized by high blood pressure within the arteries of lungs. This leads to decreased cardiac function, results in right ventricular failure that eventually causes multiorgan dysfunction and ultimately death. It is a rare disease, affecting 15 peoples per million globally with 3–5 times more common in females than males. The key hallmark histopathological manifestation of PH is pulmonary vascular remodeling, such as, medial hypertrophy, neointima formation, plexiform lesion. Pulmonary vascular remodeling is majorly driven by the dysfunction of key cellular components of the pulmonary vasculature, namely, smooth muscle, endothelial, and inflammatory cells, pericytes, and adventitial fibroblasts. Existing PAH drugs majorly dilate pulmonary arteries, increasing survival and improving quality of life, but do not target pulmonary vascular remodeling. There is a gap between our treatment and the need to reverse the pulmonary vascular remodeling to improve PAH effectively. While the exact cause of PAH is not known, genetic defects (mutations or epigenetic changes), environmental factors (e.g., hypoxia, viral infections, anorectic agents, etc.), and immune or inflammatory triggers may contribute to the cause or progression of the disease. Emerging studies suggests that lncRNAs play a significant role in pulmonary vascular and RV remodeling and PAH (Han et al., 2021).

Through a selective 84 lncRNA profiling study in hypoxic HPASMCs by a PCR array, Brock *et al.*, identified lncRNA MALAT1 as a promising candidate in PH (Brock et al., 2017). The authors demonstrated that MALAT1 was upregulated in hypoxic hPAECs and hPASMCs and in the lung of mice exposed to hypoxia. Furthermore, MALAT1 was shown to be majorly driven by HIF1a in hPAECs and hPASMCs. Silencing of MALAT1 with LNA Gapmers showed a significant inhibition of hPASMCs proliferation and migration, assessed by BrdU incorporation and wound-healing assays, respectively. Further *in vitro* studies showed that MALAT1 regulated PASMCs proliferation through targeting cyclin-dependent kinase (CDK) inhibitors. Importantly, intraperitoneal injections of LNA gapmers mediated inhibition of MALAT1 reduced heart weight in hypoxia-induced established PH in mice. Although RVSP was not changed by MALAT1 inhibition in this PH mouse model. While these findings are interesting, especially in the context of *in vivo* translation of MALAT1 findings in mice, but the authors did not able to show the direct causal effect of MALAT1 on PASMCs proliferation in mice.

Wang *et al.*, found a significantly higher expression of MALAT1 in the pulmonary arteries and hPASMCs from 8 PAH patients compared to 8 healthy control samples (Wang et al., 2019b). ShRNA-mediated MALAT1 knockdown reduced hPASMCs proliferation and migration while overexpression of the lncRNA by overexpression plasmid showed the opposite cellular phenotypes. Subsequent *in vitro* mechanistic analysis revealed that MALAT1/hsa-miR-124-3p.1/KLF5 axis plays important role in hPASMCs proliferations.

Recently, He *et al.*, also measured expression of MALAT1 and microRNA-503 by qRT-PCR in PBMCs of 45 PAH patients and 45 healthy donor controls and in HPASMCs exposed to hypoxia (He et al., 2020). Consistent with the above previous studies, the authors also found a significant upregulation of MALAT1 in PAH patients and hypoxic PASMCs. Importantly, through ROC analysis, the authors observed that MALAT1 and microRNA-503 could be used as a potential diagnostic biomarker in PAH. Additionally, the authors demonstrated that MALAT1 knockdown inhibited PASMCs proliferation and migration and induced apoptosis while overexpression studies had the opposite effects in PASMCs. MALAT1 was found to be sponged with miR-503 that controls TLR4 expression. Together, these results indicated that MALAT1 could regulate proliferation, apoptosis, and migration of PASMCs potentially through regulating miR-503/TLR4 axis. While the results of these studies are promising, there *in vitro* data need validation *in vivo* PH models as well as since all the PAH patients were recruited from only one center, their findings need to confirm in other larger cohorts of PAH patients.

## MALAT1 as a therapeutic target in lung disease

As already indicated above, one of the main challenges with lung disease management lies in the difficulty to lay hands on an effective therapeutic intervention. MALAT1 is an important lncRNA that, when extensively explored, can end our eager search for the much-anticipated breakthroughs in efficient treatments for lung diseases, making MALAT1 a potential therapeutic target for lung disease treatment. For instance, in the asthma study by Yang and Wang detailed above, the knockdown of MALAT1 reversed inflammation and mitigated bronchial and tracheal smooth muscle cell injury through the regulation of miR-133a and RyR2 (Yang and Wang, 2021). This mechanism provides the suggestion that MALAT1/miR-133a/RyR2 axis signalling network is a potential therapeutic target for asthma treatment. Also, since Huang *et al.* showed that a downregulated MALAT1, via the regulation of microRNA-216a, can significantly induce apoptosis and reduce cell proliferation, migration, and invasion, it may inform that the MALAT1/microRNA-216a signalling network can be a therapeutic target in the treatment of asthma (Huang et al., 2021). Consistently, the fact that the study by Wang *et al.* showed that an upregulated MALAT1, through interactions with miR-425 and subsequently, PTEN, was found to associate with ARDS, suggests that the MALAT1/miR-425/PTEN axis signalling network is a potential therapeutic target for ARDS treatment (Wang et al., 2019a). Lastly, since MALAT1 also plays important roles in other lung diseases some of which have been abovementioned, the following signalling networks are thought to also be key targets of therapeutic treatment for the prevention or cure of other lung diseases: MALAT1/miR-503/TLR4 (He et al., 2020) and MALAT1/hsa-miR-124-3p.1/KLF5 (Wang et al., 2019b) for PAH treatment, MALAT1/miR-125b, MALAT1/miR-146a, MALAT1/miR-133 and MALAT1/miR-203 for COPD treatment (Liu et al., 2020) and MALAT1/antisense oligonucleotides (ASOs) for lung cancer treatment (Gutschner et al., 2013). Targeting MALAT1 with RNAi approach, microRNA response elements, and ASOs could be potentially useful for developing MALAT1 targeted therapies in lung diseases.

Several drugs have also shown to improve tissue injury through targeting MALAT1 in experimental models of lung diseases. For example, by impairing the MALAT1-miR-22-3p-NLRP3 axis, resveratrol effectively blocked cardiac injury caused by pulmonary embolism (Yang et al., 2019). Suppression of MALAT1 by minocycline contributed to the amelioration of septic lung injury in ALI mice by blocking oxidative stress and inflammatory responses (Cui et al., 2021). Propofol, an intravenous anaesthetic, was shown to impedes autophagy activation and secretion of inflammatory factors (e.g., TNF-α, IL-18, and IL-1β) through reducing the MALAT1-miR-144-GSK3β signaling axis. Propofol eventually alleviated lung ischemia-reperfusion in mice (Zhang et al., 2021). Although these findings imply that the use of these drugs targeting MALAT1 could be effective in treating various lung diseases, further research is necessary to fully understand their precise mechanisms of action.

## Biomarker potential of MALAT1 in lung disease

Since MALAT1 plays important roles in the pathogenesis of lung diseases, identifying its biomarker potential would help in successful prognosis, diagnosis, and treatment of disease. Huang and colleagues asserted in their preclinical study that since MALAT1 and microRNA-216a can together regulate airway smooth muscle cell proliferation in rat models of asthma, it would be worthwhile starting to investigate both MALAT1 and microRNA-216a as potential biomarkers of asthma, and also investigate their relationship in the diagnosis and prognosis of asthma (Huang et al., 2021). MALAT1 expression is inversely associated with lung function and the Th1/Th2 ratio, and it affects GATA-3, a transcription factor that promotes the development of ILC2. ILC2, in turn, induces the biosynthesis of TH2 cytokines, such as IL-5, IL-6, and IL-13, which are implicated in the pathophysiology and clinical diagnosis of bronchial asthma (Wu et al., 2022). Poulet et al., (Poulet et al., 2020), has reported the involvement of MALAT1 in lung-related diseases. First, the authors briefly discussed about how MALAT1 dysregulation can influence the course of asthma and COPD. Now focusing on lung cancer, they mentioned in detail some of the regulators of MALAT1 (e.g., TDP-43, ESR2, Oct3/4) (Guo et al., 2015a; Jen et al., 2017; Yu et al., 2019), some genes regulated by MALAT1 (e.g., TP53, cleaved-PARP1, cleaved-CASP3, phospho-STAT3) (Tano et al., 2018; Yang et al., 2018), some miRNAs regulated by MALAT1 (e.g., hsa-miR-145, hsa-miR-204, hsa-miR-124-1) (Li et al., 2016; Li et al., 2018b; Yu et al., 2019) and MALAT1 behavior against treatment at clinical level. Given its various roles in lung cancer, the authors insisted on the need for additional studies on MALAT1 mechanisms, and their confirmation as potential biomarkers through further research. Liu and colleagues also found that upregulation of MALAT1, as well as regulation of its targets miR-125b, miR-146a, and miR-203, are important for detecting increased risk of COPD or for managing COPD (Liu et al., 2020). Patients with lung cancer have lower levels of MALAT1 in their blood compared to healthy individuals, with an area under the receiver operator curve of 0.718 (Guo et al., 2015b). The expression of MALAT1 was found to be stronger in the whole blood of lung cancer patients with metastasis compared to those without metastasis. As MALAT1 can be detected in whole blood, has the potential to be a useful biomarker for identifying and facilitating the treatment of lung cancer. MALAT1 is also involved in various other lung diseases as detailed in earlier sections of this review. In summary, with these clues, it is worthwhile to start thinking about MALAT1 is a potential biomarker for lung diseases.

## Concluding remark and future direction

MALAT1 has recently become one of the most studied lncRNAs for genetic, epigenetic, and molecular research due to their various impacts on health and diseases. They are efficient in facilitating vital processes such as gene regulation, upholding of physiological conditions (Ma et al., 2015), and their help to maintain normal ageing process (Lei et al., 2017) and immunity (Sookoian et al., 2018). Through interaction with target proteins such as miRNAs, MALAT1 can alleviate lung disease progression, or at least mitigate it. However, they can become toxic and drive disease pathogenesis and progression when aberrant, because of dysregulation. This evidence suggests that MALAT1 is an important entity in lung disease study and management, and it has the potential of being both a reliable biomarker in lung diseases and therapeutic target for lung disease treatment. Current and future researchers and/or clinicians can capitalize on this for an improved research and treatment of lung diseases. However, to achieve this, additional work has to be done, which relates to addressing some of the limitations indicated in previous studies on MALAT1 and lung disease relationships**.**
